# Supplementary material for: Structure of the Dual-Mode Wnt Regulator Kremen1 and Insight into Ternary Complex Formation with LRP6 and Dickkopf
Source: Structure. 2016 Sep 6;24(9):1599–605. doi: 10.1016/j.str.2016.06.020 (PMC5014086; doi:10.1016/j.str.2016.06.020)
Supplement: Document S1. Figure S1 [file mmc1.pdf]

Structure, Volume *24*

Supplemental Information

Structure of the Dual-Mode Wnt Regulator Kremen1 and Insight into Ternary Complex Formation with LRP6 and Dickkopf

Matthias Zebisch, Verity A. Jackson, Yuguang Zhao, and E. Yvonne Jones

Structure of the dual mode Wnt regulator Kremen1 and insight into ternary complex formation with LRP6 and Dickkopf

Matthias Zebisch, Verity A. Jackson, Yuguang Zhao & E. Yvonne Jones

Supplementary Information

Inventory of Supplemental Information

Figure S1: Representative electron density for the ternary complex solved at low resolution. Relates to Figure 2.

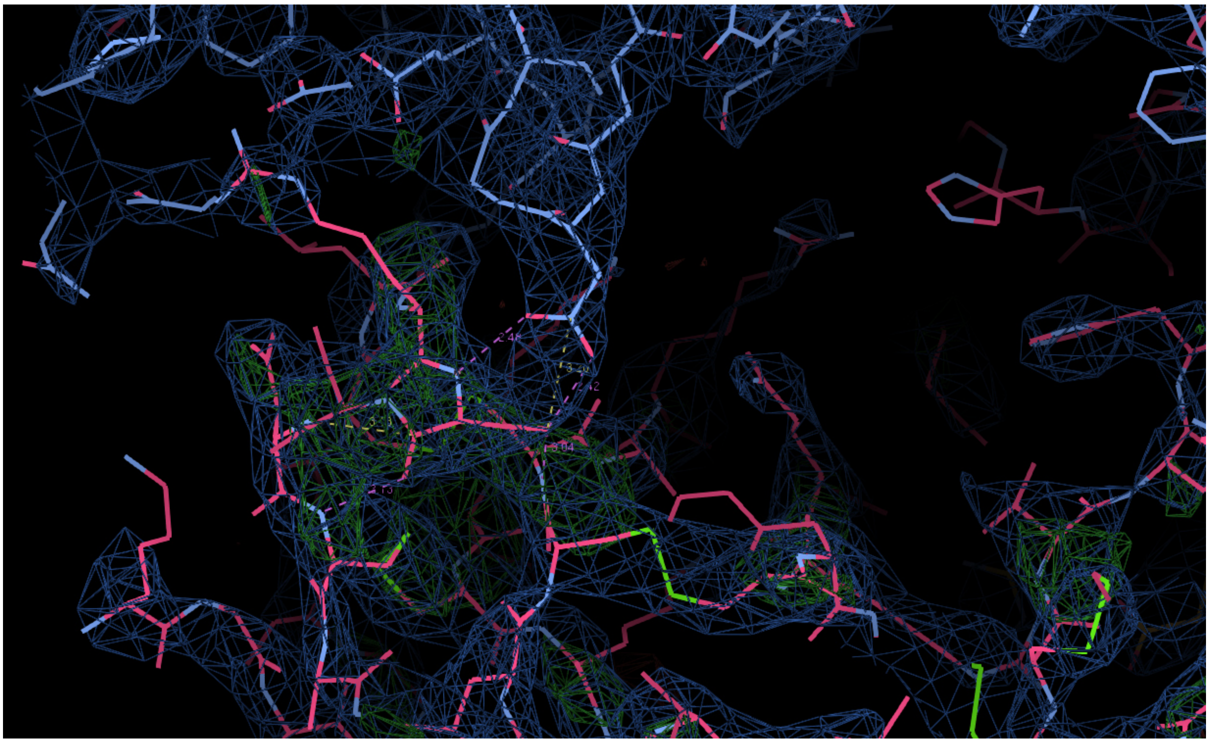

Figure S1: Representative electron density for the ternary complex solved at low resolution. Relates to Figure 2. The KRM1-DKK1 interaction around D201 (KRM1) and S192 (DKK1) is highlighted. Shown is the 2FoFc map calculated for the final model contoured at 1σ in blue and the FoFc DKK1 omit map contoured at 3σ in green.
