# Supplementary material for: Structure of the Dual-Mode Wnt Regulator Kremen1 and Insight into Ternary Complex Formation with LRP6 and Dickkopf
Source: Structure. 2016 Sep 6;24(9):1599–605. doi: 10.1016/j.str.2016.06.020 (PMC5014086; doi:10.1016/j.str.2016.06.020)
Supplement: Document S2. Article plus Supplemental Information [file mmc2.pdf]

Short Article

Structure

Structure of the Dual-Mode Wnt Regulator Kremen1 and Insight into Ternary Complex Formation with LRP6 and Dickkopf

Graphical Abstract

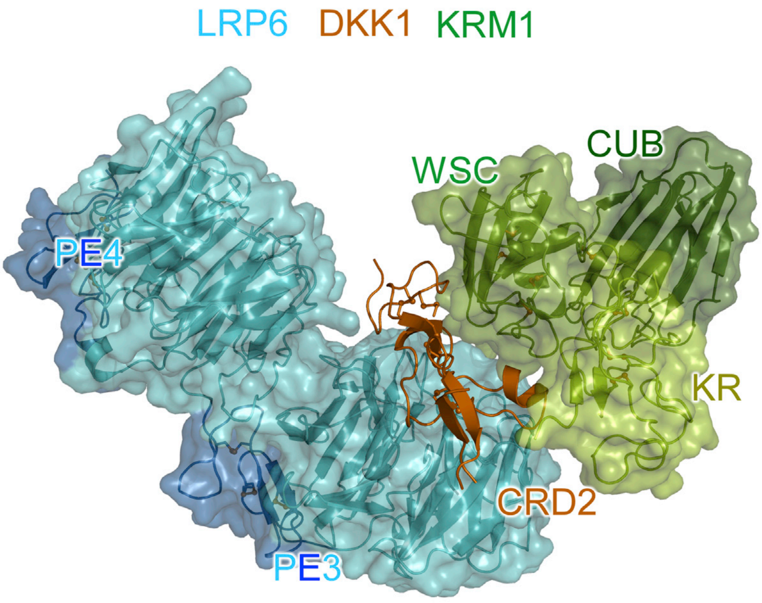

Authors

Matthias Zebisch, Verity A. Jackson, Yuguang Zhao, E. Yvonne Jones

Correspondence

matthias.zebisch@evotec.com (M.Z.), yvonne@strubi.ox.ac.uk (E.Y.J.)

In Brief

Zebisch et al. describe the ectodomain structure of KREMEN 1, a receptor for Wnt antagonists of the DKK family. Apo structures and a complex with functional fragments of DKK1 and LRP6 shed light on the function of this dual-mode regulator of Wnt signaling.

Highlights

- The structure of the KREMEN 1 ectodomain is solved from three crystal forms
- Kringle, WSC, and CUB subdomains interact tightly to form a single structural unit
- The interface to DKKs is formed from the Kringle and WSC domains
- The CUB domain is found to interact directly with $LRP6_{PE1PE2}$

Accession Numbers

5FWS
5FWT
5FWU
5FWV
5FWW

Zebisch et al., 2016, Structure *24*, 1599–1605
September 6, 2016 © 2016 The Authors. Published by Elsevier Ltd.
http://dx.doi.org/10.1016/j.str.2016.06.020

Structure
Short Article

Structure of the Dual-Mode Wnt Regulator Kremen1 and Insight into Ternary Complex Formation with LRP6 and Dickkopf

Matthias Zebisch,[1,2,*] Verity A. Jackson,[1,3] Yuguang Zhao,[1] and E. Yvonne Jones[1,*]
[1]Division of Structural Biology, Wellcome Trust Centre for Human Genetics, University of Oxford, Oxford OX3 7BN, UK
[2]Present address: Evotec (UK) Ltd., 114 Innovation Drive, Milton Park, Abingdon, Oxfordshire OX14 4RZ, UK
[3]Present address: Department of Biochemistry, Oxford University, South Parks Road, Oxford OX1 3QU, UK
*Correspondence: matthias.zebisch@evotec.com (M.Z.), yvonne@strubi.ox.ac.uk (E.Y.J.)
http://dx.doi.org/10.1016/j.str.2016.06.020

SUMMARY

Kremen 1 and 2 have been identified as co-receptors for Dickkopf (Dkk) proteins, hallmark secreted antagonists of canonical Wnt signaling. We present here three crystal structures of the ectodomain of human Kremen1 (KRM1$_{ECD}$) at resolutions between 1.9 and 3.2 Å. KRM1$_{ECD}$ emerges as a rigid molecule with tight interactions stabilizing a triangular arrangement of its Kringle, WSC, and CUB structural domains. The structures reveal an unpredicted homology of the WSC domain to hepatocyte growth factor. We further report the general architecture of the ternary complex formed by the Wnt co-receptor Lrp5/6, Dkk, and Krm, determined from a low-resolution complex crystal structure between β-propeller/EGF repeats (PE) 3 and 4 of the Wnt co-receptor LRP6 (LRP6$_{PE3PE4}$), the cysteine-rich domain 2 (CRD2) of DKK1, and KRM1$_{ECD}$. DKK1$_{CRD2}$ is sandwiched between LRP6$_{PE3}$ and KRM1$_{Kringle\text{-}WSC}$. Modeling studies supported by surface plasmon resonance suggest a direct interaction site between Krm1$_{CUB}$ and Lrp6$_{PE2}$.

INTRODUCTION

Signaling by Wnt morphogens is renowned for its fundamental roles in embryonic development, tissue homeostasis, and stem cell maintenance (Holstein, 2012). Due to these functions, generation, delivery, and interpretation of Wnt signals are all heavily regulated in the animal body (Clevers and Nusse, 2012; Niehrs, 2012; Malinauskas and Jones, 2014; Kakugawa et al., 2015). Vertebrate Dickkopf proteins (Dkk1, 2, and 4) are one of many secreted antagonists of Wnt and function by blocking access to the Wnt co-receptor LRP5/6 (Semenov et al., 2001; Mao et al., 2001; Bafico et al., 2001). Kremen proteins (Krm1 and Krm2) have been identified as additional high-affinity transmembrane receptors for Dkk (Nakamura et al., 2001, 2008; Mao et al., 2002). Krm and Dkk synergize in Wnt inhibition during *Xenopus* embryogenesis to regulate anterior-posterior patterning (Davidson et al., 2002). Mechanistically it is thought that, in the presence of Dkk, Krm forms a ternary complex with Lrp6, which is then rapidly endocytosed. This amplifies the intrinsic Wnt antagonistic activity of Dkk by efficiently depleting the cell surface of the Wnt co-receptor (Mao et al., 2002). In accordance with this, *Krm1*$^{-/-}$ and *Krm2*$^{-/-}$ double knockout mice show a high bone mass phenotype typical of increased Wnt signaling, as well as growth of ectopic forelimb digits. Growth of ectopic digits is further enhanced upon additional loss of *dkk* expression (Ellwanger et al., 2008; Schulze et al., 2010). The Wnt antagonistic activity of Krm1 is also linked to its importance for correct thymus epithelium formation in mice (Osada et al., 2006). The importance of intact KRM1 for normal human development and health is highlighted by the recent finding that a homozygous mutation in the ectodomain of KRM1 leads to severe ectodermal dysplasia including oligodontia (Issa et al., 2016). Interestingly, the Wnt antagonistic activity of Krm is context dependent, and Krm proteins are actually dual-mode Wnt regulators. In the absence of Dkk, Krm1 and 2 change their function from inhibition to enhancement of Lrp6-mediated signaling. By direct binding to Lrp6 via the ectodomains, Krm proteins promote Lrp6 cell-surface localization and hence increase receptor availability (Hassler et al., 2007; Cselenyi and Lee, 2008). Further increasing the complexity of Krm functionality, it was recently found that Krm1 (but not Krm2) can also act independently of LRP5/6 and Wnt as a dependence receptor, triggering apoptosis unless bound to Dkk (Causeret et al., 2015).

Structurally, Krm1 and 2 are type I transmembrane proteins with a 40 kDa ectodomain and a flexible cytoplasmic tail consisting of 60–75 residues. The ectodomain consists of three similarly sized structural domains of around 10 kDa each: the N-terminal Kringle domain (KR) is followed by a WSC domain of unknown fold (Verna et al., 1997). The third structural domain is a CUB domain (Romero et al., 1997). An approximately 70-residue linker connects the CUB domain to the transmembrane span. An intact KR-WSC-CUB domain triplet and membrane attachment is required for Wnt antagonism (Mao et al., 2002). The transmembrane span and cytoplasmic tail can be replaced with a GPI linker without impact on Wnt antagonism (Mao et al., 2002; Causeret et al., 2015).

We sought to provide structural insights into the multifunctionality of this cell-surface receptor. The structures presented here reveal the unknown fold of the WSC domain and the tight interactions of all three domains. We further

This is an open access article under the CC BY license (http://creativecommons.org/licenses/by/4.0/).

Table 1. Diffraction and Refinement Statistics

	$KRM1_{ECD}$	$KRM1_{ECD}$	$KRM1_{ECD}$	$KRM1_{ECD}$	$LRP6_{PE3PE4}$-DKK_{CRD2}-$KRM1_{ECD}$
Crystal form	I	I	II	III	I
X-ray source	Diamond i04	Diamond i03	Diamond i03	Diamond i04	Diamond i04
Wavelength (Å)	0.9793	0.9700	0.9700	0.9795	0.9795
Space group	$P3_121$	$P3_121$	$P4_3$	$P4_12_12$	$C222_1$
Unit cell a/α (Å/°)	50.9/90	50.5/90	65.8/90	67.8/90	86.9/90
b/β (Å/°)	50.9/90	50.5/90	65.8/90	67.8/90	100.1/90
c/γ (Å/°)	188.4/120	187.4/120	75.0/90	198.2/90	270.7/90
Wilson B factor ($Å^2$)	31	41	76	77	NA
Resolution range (Å)	47.10–1.90 (1.95–1.90)	62.47–2.10 (2.16–2.10)	75.00–2.80 (2.99–2.80)	67.80–3.20 (3.42–3.20)	67.68–3.50 (7.16–6.40, 3.92–3.50)
Unique reflections	23,300 (1,524)	17,089 (1,428)	7,964 (1,448)	8,171 (1,343)	8,070 (723, 645)
Average multiplicity	9.1 (9.2)	5.2 (5.3)	3.7 (3.7)	22.7 (12.6)	3.8 (3.5, 4.4)
Completeness (%)	99.8 (98.5)	100 (100)	99.8 (100)	98.8 (93.4)	51.6 (98.5, 14.1)
$\langle I/\sigma I \rangle$	11.4 (1.7)	12.0 (1.7)	14.9 (1.5)	13.1 (1.9)	4.6 (4.1, 2.2)
R_{merge} (%)	14.8 (158.3)	9.3 (98.0)	6.2 (98.9)	29.8 (142.2)	44.9 (40.5, 114.2)
R_{pim} (%)	15.7 (55.3)	10.3 (109.0)	3.7 (53.8)	6.3 (40.0)	24.7 (23.9, 59.9)
Refinement					
R_{work} (%)	17.9	18.4	21.6	20.2	32.1
R_{free} (%)	22.7	23.2	30.7	27.1	35.5
No. of Non-Hydrogen Atoms					
Protein	2,260	2,301	2,102	2,305	7,730
N-glycans	42	42	28	28	0
Water	79	54	0	2	0
Ligands	6	6	2	5	0
Average B factor ($Å^2$)					
Protein	63	65	108	84	–
N-glycans	35	46	102	18	–
Water	68	85	–	75	–
Ligands	36	47	91	75	66
RMSD from Ideality					
Bond lengths (Å)	0.020	0.016	0.019	0.016	0.004
Bond angles (°)	2.050	1.748	1.952	1.796	0.770
Ramachandran Plot					
Favored (%)	96.8	95.5	96.9	94.9	92.3
Allowed (%)	99.7	100.0	100.0	99.7	99.8
Number of outliers	1	0	0	1	2
PDB code	5FWS	5FWT	5FWU	5FWV	5FWW

Values in parentheses refer to the highest-resolution shell. An additional shell given for the ternary complex corresponds to the last shell with near-complete diffraction data. NA, not announced.

succeeded in determination of a low-resolution $LRP6_{PE3PE4}$-$DKK1_{CRD2}$-$KRM1_{ECD}$ complex, defining the architecture of the Wnt inhibitory complex that leads to Lrp6 cell-surface depletion.

RESULTS

The recombinant production of the extracellular domain of Krm for structural studies proved challenging (see Experimental Procedures). We succeeded in purifying $KRM1_{ECD}$ complexes with $DKK1_{fl}$, $DKK1_{Linker\text{-}CRD2}$, and $DKK1_{CRD2}$ that were monodisperse and stable in gel filtration, hence indicating at least micromolar affinity (data not shown). Several crystal forms were obtained from these complexes, however, crystals always contained only KRM1 protein.

We solved the structure of $KRM1_{ECD}$ in three crystal forms at 1.9, 2.8, and 3.2 Å resolution (Table 1). The high-resolution structure is a near full-length model (Figure 1). The small, flexible, and charged ^{98}AEHED^{102} loop could only be modeled in a slightly lower resolution structure and in crystal form III. The KR, WSC, and CUB are arranged in a roughly triangular fashion with tight interactions between all three domains. The KR domain,

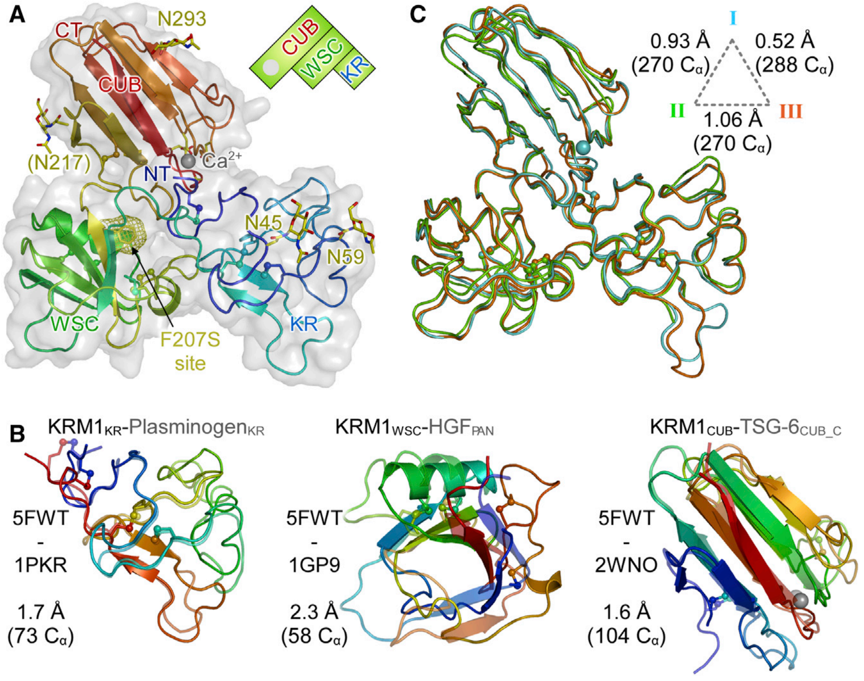

Figure 1. Structure of Unliganded $KRM1_{ECD}$

(A) The $KRM1_{ECD}$ fold (crystal form I) colored blue to red from the N to C terminus. Cysteines as ball and sticks, glycosylation sites as sticks. The bound calcium is shown as a gray sphere. The site of the F207S mutation associated with ectodermal dysplasia in humans is shown as mesh.

(B) Superposition of the three $KRM1_{ECD}$ subdomains (solid) with their next structurally characterized homologs (half transparent).

(C) Superposition of $KRM1_{ECD}$ from the three crystal forms. Alignment scores for each pairing are indicated on the dashed triangle.

which bears two of the four glycosylation sites, contains the canonical three disulfide bridges (C32-C114, C55-C95, C84-C109) and, like other Kringle domains, is low in secondary structure elements. The structurally most similar Kringle domain is that of human plasminogen (PDB: 1PKR; Wu et al., 1994) with an root-mean-square deviation (RMSD) of 1.7 Å for 73 aligned C_α (Figure 1B). The KRM1 structure reveals the fold of the WSC domain for the first time. The structure is best described as a sandwich of a β1-β5-β3-β4-β2 antiparallel β sheet and a single α helix. The structure is also rich in loops and is stabilized by four disulfide bridges (C122-C186, C147-C167, C151-C169, C190-C198). Using the PDBeFold server, we detected a surprising yet significant homology to PAN module domains (Tordai et al., 1999). The closest structural relative is hepatocyte growth factor (HGF, PDB: 1GP9; Watanabe et al., 2002), which superposes with an RMSD of 2.3 Å for 58 aligned C_α (Figure 1B). The CUB domain bears two glycosylation sites. Although present, the quality of the electron density around N217 did not allow modeling of the sugar moiety. In crystal form I, a calcium ion is present at the canonical position (Gaboriaud et al., 2011) coordinated by the carboxylates of D263, D266 (bidentate), and D306, as well as the carbonyl of N309 and a water molecule. The coordination sphere deviates significantly from perfectly octahedral (not shown). This might result in the site having a low affinity and may explain why calcium is not present in the two low-resolution crystal forms. Loss of calcium has led to loop rearrangements and partial disorder in these crystal forms. The closest structural relative is the CUB_C domain of Tsg-6 (PDB: 2WNO; Briggs et al., 2015), which superposes with KRM_{CUB} with an RMSD of 1.6 Å for 104 C_α (Figure 1B).

A superposition of the three KRM1 structures reveals no major structural differences (Figure 1C) as anticipated from the plethora of interactions between the three domains. Minor differences are caused by the collapse of the Ca^{2+} binding site in crystal forms II and III and loop flexibility in the KR domain. The F207S mutation recently found to cause ectodermal dysplasia in Palestinian families (Issa et al., 2016) maps to the hydrophobic core of the protein at the interface of the three subdomains (Figure 1A). Such a mutation is bound to severely destabilize the protein structure of KRM1, leading to disturbance of its Wnt antagonistic, Wnt stimulatory, and Wnt independent activity.

Low-Resolution Insight into Ternary Complex Formation

Co-crystallization of $LRP6_{PE3PE4}$ with $DKK1_{CRD2}$, and $LRP6_{PE1}$ with an N-terminal peptide of DKK1 has provided valuable structural insight into direct Wnt inhibition by Dkk ligands (Cheng et al., 2011; Ahn et al., 2011; Bourhis et al., 2011; Bao et al., 2012). One face of the rather flat $DKK1_{CRD2}$ fragment binds to the third β propeller of LRP6. Mutational analyses further implied that the $LRP6_{PE3}$-averted face of $DKK1_{CRD2}$ bears the Krm binding site, hence suggesting how Dkk can recruit both receptors into a ternary complex (Wang et al., 2008).

To obtain direct insight into ternary complex formation by Lrp5/6, Dkk, and Krm, we subjected an $LRP6_{PE3PE4}$-$DKK1_{fl}$-$KRM1_{ECD}$ complex to crystallization trials. Diffraction data collected from the resulting crystals were highly anisotropic with diffraction extending in the best directions to 3.5 Å and 3.7 Å but only to 6.4 Å in the third direction. Despite the lack of high-resolution diffraction, the general architecture of the ternary complex is revealed (Figure 2A). $DKK1_{CRD2}$ binds to the top face of the LRP6 PE3 β propeller as described earlier for the binary complex (Cheng et al., 2011; Ahn et al., 2011). $KRM1_{ECD}$ does indeed bind on the opposite side of $DKK1_{CRD2}$ with only its KR and WSC domains engaged in binding (Figure 2A). Although present in the complex subjected to crystallization, we observe no density that could correspond to CRD1 or the domain linker (L). We confirm that the CRD2 of DKK1 is required and sufficient for binding to KRM1 (Mao and Niehrs, 2003): In surface plasmon resonance (SPR), we measured low micromolar affinity between full-length DKK1 and immobilized $KRM1_{ECD}$ (Figure 2B). A SUMO fusion of $DKK1_{L\text{-}CRD2}$ displayed a similar (slightly higher) affinity. In contrast, a SUMO fusion of $DKK1_{CRD1\text{-}L}$ did not display binding for concentrations tested up to 325 μM (Figure 2B).

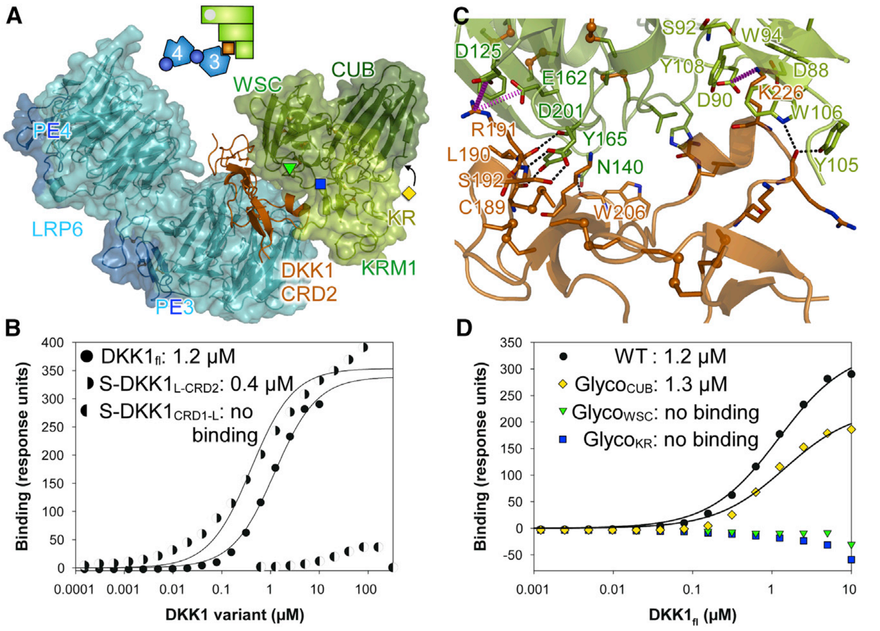

Figure 2. Insight into Ternary Complex Formation

(A) The structure of the ternary $LRP6_{PE3PE4}$-$DKK1_{CRD2}$-$KRM1_{ECD}$ complex. DKK1 (orange) is sandwiched between the PE3 module of LRP6 (blue) and the KR-WSC domain pair of KRM1 (green). Colored symbols indicate introduced N-glycan attachment sites (see D).

(B) SPR data comparing binding of full-length DKK1 and SUMO fusions of DKK1 truncations for binding to immobilized wild-type $KRM1_{ECD}$.

(C) Close-up view of the $DKK1_{CRD2}$-$KRM1_{ECD}$ interface. Residues involved in interface formation are shown as sticks; those mentioned in the text are labeled. Salt bridges are in pink and hydrogen bonds in black. Model bias cannot be excluded as single atoms and bonds are not resolved at 6.4–3.5 Å. See also Figure S1.

(D) SPR binding data comparing DKK1 analyte binding with wild-type $KRM1_{ECD}$ and three variants bearing engineered glycosylation sites on the KR and WSC domains (green and blue pointing to DKK1) and on the CUB domain (orange). See also symbols in (A).

Overall, the DKK1-KRM1 interface is characterized by a large number of polar interactions but only few hydrophobic contacts (Figure 2C). The crystal structure gives an explanation for DKK1 loss-of-binding mutations identified previously (Wang et al., 2008): R191 of DKK1 forms a double salt bridge to D125 and E162 of KRM1 (Figure 2C). A charge reversal as in the mouse Dkk1 (mDkk1) R197E variant would severely disrupt the binding. Similarly, the K226 side chain of DKK1, which points to a small hydrophobic pocket on the surface of KRM1 formed by Y108, W94, and W106, forms salt bridges with the side chains of KRM1 D88 and D90. Again, a charge reversal as shown before for mDkk1 K232E would be incompatible with binding. The side chain of DKK1 S192 was also predicted to be involved in Krm binding (Wang et al., 2008). Indeed, we found (Figure 2C) that the side chain of D201 of KRM1 forms two hydrogen bonds to the side-chain hydroxyl and the backbone amide of S192 (mouse, S198). Additional polar interactions are formed between the N140, S163, and Y165 side chains of KRM1 and DKK1 backbone carbonyls of W206, L190, and C189, respectively. The carbonyl of DKK1 R224 is hydrogen bonded to Y105 and W106 of KRM1. We suspect that the Dkk charge reversal mutations performed in the murine background and shown to diminish Krm binding K211E and R203E (mouse K217E and R209E; Chen et al., 2008) do so likely indirectly by disruption of the Dkk fold. We further validated the DKK1 binding site on KRM1 by introducing glycosylation sites at the KR (^{90}DVS^{92} → NVS) and WSC (^{189}VCF^{191} → NCS) domains pointing toward DKK (Figures 2A and 2D). Introduction of N-linked glycans in protein-protein-binding sites is an established way of disrupting protein-binding interfaces. Both ectodomain mutants were secreted comparably with the wild-type, indicating correct folding, but failed to achieve any detectable binding in SPR using full-length DKK1 as analyte. In contrast, a mutant carrying an additional N-glycan outside the interface at the CUB domain (^{309}NQA^{311} → NQS), was wild-type-like in DKK1 binding (Figure 2D).

Identification of a Direct LRP6-KRM1 Binding Site

The $LRP6_{PE3PE4}$-$DKK1_{CRD2}$-$KRM1_{ECD}$ complex structure reveals no direct interactions between KRM1 and LRP6. We constructed in silico a ternary complex with a close to full-length LRP6 ectodomain (PE1PE2PE3PE4 horse shoe) similar to Chen et al. (2011) but without refinement against electron microscopy (EM) or small-angle X-ray scattering data. An auxiliary PE3PE4 fragment was superimposed via PE4 onto PE3 of the crystal structure, and the $LRP6_{PE1PE2}$ structure was superimposed via PE2 onto PE3 of this auxiliary fragment (Figure 3A).

For this crude approximation of a true ternary complex, we noted very close proximity between the Ca^{2+}-binding region of KRM1 and the top face of the PE2 β propeller of LRP6. The solvent-exposed residues R307, I308, and N309 of the central Ca^{2+}-binding β connection loop of KRM1 would be almost ideally positioned for binding to this face, which is commonly used as a binding site on β propellers. Peptides containing arginine/lysine, isoleucine, and asparagine (consensus sequence N-X-I-(G)-R/K; Bourhis et al., 2011) are also employed by DKK1 and SOST to bind to LRP6 (albeit to propeller 1; Figure 3B). To support the hypothesis that $KRM1_{CUB}$ binds to $LRP6_{PE2}$, we used SPR and compared binding of the wild-type and the GlycoCUB mutant of $KRM1_{ECD}$ (bearing an N-glycosylation site at N309) with a purified $LRP6_{PE1PE2}$ fragment. Indeed, we found that in the absence of Dkk, $KRM1_{ECD}$ bound with considerable affinity to $LRP6_{PE1PE2}$ (Figure 3C). In contrast, no saturable binding was observed between KRM1 and $LRP6_{PE3PE4}$. Introduction of an N-glycosylation site at N309 in $KRM1_{ECD}$ abolished $LRP6_{PE1PE2}$ binding (Figure 3C), while binding to DKK1 was unaffected (Figure 2D). We conclude that the predicted binding site between $KRM1_{CUB}$ and $LRP6_{PE2}$ is a strong candidate for mediating the direct Lrp6-Krm interaction, which is thought to increase Wnt responsiveness by stabilizing Lrp6 at the cell surface (Hassler et al., 2007; Cselenyi and Lee, 2008). Further experiments are required to pinpoint the exact binding site. Although $LRP6_{PE1}$ appears somewhat out of reach in the modeled ternary complex, it cannot

CellPress

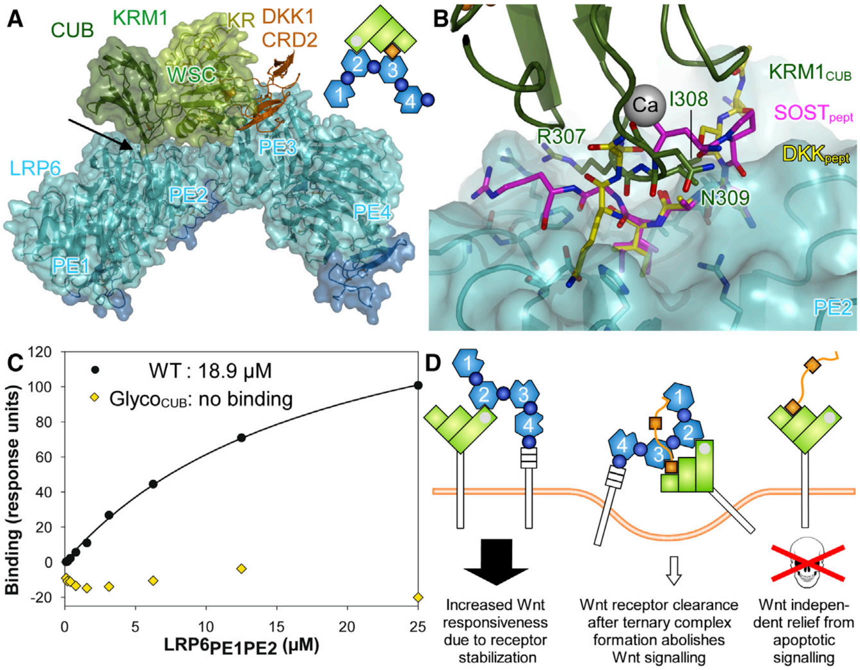

Figure 3. LRP6-KRM1 Direct Interaction and Summary

(A) In a construction of a ternary complex with all four β propellers of LRP6 intact, the CUB domain points via its Ca^{2+}-binding region toward the top face of the second β propeller.

(B) Close-up view of the potential interaction site. In addition, $LRP6_{PE2}$ has been superimposed with DKK1 (yellow) and SOST (pink) peptide complexes of $LRP6_{PE1}$.

(C) SPR measurements comparing $LRP6_{PE1PE2}$ binding with wild-type $KRM1_{ECD}$ and the $Glyco_{CUB}$ mutant bearing an N-glycan at N309.

(D) Schematic representation of structural and biophysical findings and their implications for Wnt-dependent (left, middle) and independent (right) signaling. Conformational differences in the depictions of LRP6 are included purely for ease of representation.

be excluded as the Krm binding site in the ternary complex and LRP6-Krm binary complex. The presence of DKK may govern which propeller (PE1 versus PE2) of LRP6 is available for Krm binding.

Apparent binding across the proposed $KRM1_{CUB}$-$LRP6_{PE2}$ interface is expected to be higher once Krm is also cross-linked to $LRP6_{PE3}$ via $DKK1_{CRD2}$ (Figure 3D). Low-resolution negative-stain EM and small-angle X-ray scattering studies of $LRP6_{PE1PE2PE3PE4}$, in isolation and in complex with Dkk1 (Cheng et al., 2011; Ahn et al., 2011), plus negative-stain EM of full-length LRP6 ectodomain (Chen et al., 2011), have indicated curved, platform-like conformations but also potential flexibility between PE2 and PE3. It is therefore possible that the interplay of Krm and Dkk binding can promote changes in LRP6 ectodomain conformation with functional consequences; however, such ideas await investigation.

Taken together, the structural and biophysical studies we report here extend our mechanistic understanding of Wnt signal regulation. We describe the ectodomain structure of the dual Wnt regulator Krm1, providing an explanation for the detrimental effect on health and development of a homozygous KRM1 mutation. We also reveal the interaction mode of Krm-Dkk and the architecture of the ternary complex formed by Lrp5/6, Dkk, and Krm. Furthermore, the ternary crystal structure has guided in silico and biophysical analyses to suggest a direct LRP6-KRM1 interaction site. Our findings provide a solid foundation for additional studies to probe how ternary complex formation triggers internalization, whereas Krm binding in the absence of Dkk stabilizes the Wnt co-receptor at the cell surface.

EXPERIMENTAL PROCEDURES

Large-Scale Mammalian Expression and Protein Purification

Krm_{ECD} fragments were cloned into pHLsec or variants thereof (Seiradake et al., 2015). Full ectodomain variants (e.g., KRM1 isoform 3, P30-T377) were well secreted into the conditioned medium (CM) of HEK293T cells, but exhibited extensive O-glycosylation (as judged from smeary bands in western blot), which would be detrimental to crystallization. Fragments truncated to the KR-WSC-CUB core gave sharp bands but were barely secreted. We therefore engineered an A23-G373 (isoform 1 numbering used throughout the article) full ectodomain construct ($KRM1_{ECD\text{-}TEV}$) with a C-terminal His10 tag that contained a TEV protease cleavage site after E324. The expected sequence of the secreted protein is ETG-[23]APSPGLGPGPE[31] … [320]AVKEE[324]-GSENLYFQGGS-[325]LPQ … VPG[373]-THHHHHHHHHH (the isoform-2-specific PG insertion and the TEV site are underlined). This construct was well secreted and could be processed using TEV protease. However, 80%–90% of the protein eluted as aggregates from a size-exclusion column even before TEV treatment. The same applied to analog constructs for Krm1 from zebrafish, frog, and mouse. No monomeric protein at all could be obtained for several Krm2 constructs from multiple species. A $KRM1_{ECD\text{-}TEV}$ expressing stable GntI-deficient HEK293S cell line was generated by excision of an *Eco*RI-*Xho*I fragment, sub-cloning into pNeo-Sec-1, and selection of neomycin-resistant cells (Seiradake et al., 2015). The stable cell line showed expression levels superior to transiently transfected cells (not shown).

Human $LRP6_{PE1PE2}$, $LRP6_{PE3PE4}$, and full-length DKK1 were produced in a similar way as described (Chen et al., 2011). Shorter constructs of DKK1 lacking the N-terminal flexible region and CRD1 were not secreted from HEK cells. However, using the approach of an N-terminal fusion to a modified SUMO protein as described earlier (Peroutka et al., 2008; Chang et al., 2015), we succeeded in secretory expression of a SUMO-$DKK1_{Linker\text{-}CRD2}$ construct encompassing residues S141-H266. A variant of this containing a TEV cleavage site just before T181, SUMO-$DKK1_{Linker\text{-}TEV\text{-}CRD2}$, was also well expressed and allowed removal of the flexible linker region.

To obtain complexes of $KRM1_{ECD\text{-}TEV}$, we (co-)transfected the stable cell line with DKK and $LRP6_{PE3PE4}$ constructs described earlier (Chen et al., 2011). Binary and ternary $KRM1_{ECD}$-$DKK1_{fl}$ and $KRM1_{ECD}$-$DKK1_{fl}$-$LRP6_{PE3PE4}$ complexes were stable in gel-filtration eluting as distinct monodisperse peaks.

Crystallization and Data Collection

All samples subjected to crystallization were purified from CM by affinity and size-exclusion chromatography (Zebisch et al., 2013; Kakugawa et al., 2015). After treatment with TEV protease and endoglycosidase F1 overnight using mass equivalents of 1%, samples were subjected to size-exclusion chromatography in 10 mM HEPES/NaOH (pH 7.5), 150 mM NaCl. The crystals giving rise to the 1.9 Å dataset for KRM1 in crystal form I were obtained from a $KRM1_{ECD}$-$DKK1_{Linker\text{-}CRD2}$ complex concentrated to 12 mg/mL. Out of this complex, $KRM1_{ECD}$ crystallized alone in 2.0 M ammonium sulfate, 5% (v/v) iso-propanol. For cryoprotection, crystals were transferred to mother liquor

mixed 1:1 with 3.4 M sodium malonate (pH 7.0). The slightly less well-ordered crystal of crystal form I and crystals of form II were obtained from a $KRM1_{ECD}$-$DKK1_{CRD2}$ complex using the SUMO-$DKK1_{Linker\text{-}TEV\text{-}CRD2}$ construct and releasing SUMO and the DKK linker region by TEV and 3C protease treatment. Crystals of form I (2.1 Å) appeared from protein at 12 mg/mL in 1.0 M $(NH_4)H_2PO_4$, 0.100 M sodium citrate (pH 5.6) and were cryoprotected by transfer to 2.9 M sodium malonate (pH 5.0). Crystals of form II grew from protein concentrated to 17 mg/mL in 1.0 M $MgSO_4$, 0.1 M trisodium citrate (final pH 5.6). For cryoprotection, crystals were transferred to mother liquor mixed 1:3 with 3.0 M ammonium sulfate, 18% glycerol. Crystal form III appeared after 11 months in a dried-out drop of condition H5 of the Morpheus screen. The protein concentration had been 9 mg/mL. For cryoprotection, fresh liquid from Morpheus/H5 was added. The ternary complex structure was obtained from an $LRP6_{PE3PE4}$-$DKK1_{fl}$-$KRM1_{ECD}$ complex at 9 mg/mL that grew in condition E10 of the PACTpremier screen (pH approximately 6.8) over the course of 2–11 months. For cryoprotection, 10% PEG200 was added. By mistake, the crystals were incubated for 1 hr with 1 mM platinum compound in this cryosolution before cryocooling.

Structure Determination

Diffraction data were collected at DIAMOND synchrotron light source at the beamlines detailed in Table 1. The structure was initially solved from crystal form III by molecular replacement (MR) with PHASER (McCoy et al., 2007), placing models for the CUB domain (PDB: 2WNO, CUB_C domain of Tsg-6 (Briggs et al., 2015), 37% sequence identity), and the KR domain (PDB: 1PKR, Kringle 1 of plasminogen; Wu et al., 1994; 39% sequence identity). Traceable density for the WSC domain became immediately evident. The KRM1 structure was then built and refined by cycling between the various crystal forms.

For the ternary complex, we obtained only a low-resolution, highly anisotropic dataset extending to Bragg spacings of 3.5 Å, 6.4 Å, and 3.7 Å along the three principle directions ($<I/\sigma I> = 2$). All data to 3.5 Å were used during structure determination by MR. $LRP6_{PE3PE4}$ (PDB: 4A0P; Chen et al., 2011) and $KRM1_{ECD}$ (both stripped of glycosylation sites) could be placed independently by PHASER, giving Z scores of >10 and log likelihood gains (LLG) of >200. The combined LLG was 673, increasing to 901 after rigid-body refinement. Strong electron density became apparent at glycosylation sites and close to methionines (see platinum soak above), further supporting the MR solution. Additional strong density was evident between LRP6 and KRM1, suggesting the presence of DKK1. A model of the $DKK1_{CRD2}$ (PDB: 3S2K and 3S8V (Cheng et al., 2011; Ahn et al., 2011)) could then be placed with PHASER by testing all rotation function peaks. This increased the LLG from 901 to 973 indicating a correct solution. The individually placed LRP6 and DKK models were then replaced with chains B and C from the LRP6-DKK complex in PDB: 3S2K. The structure was subjected to rigid-body refinement using single structural domains as individually positioned bodies.

We then performed restrained refinement of the coordinates against the ellipsoidally truncated and anisotropically scaled (Strong et al., 2006) diffraction data as obtained from the diffraction anisotropy server at UCLA. The resolution cutoffs were 3.5 Å, 6.4 Å, and 3.7 Å. Strong geometric restraints generated by PROSMART from the available high-resolution reference structures were used during refinement. No manual model building was attempted. Restrained refinement was followed by ten cycles of structure idealization. The final model had R_{work}/R_{free} errors of 32.5%/36.1% against the anisotropy-corrected data and 32.1%/35.5% against the unmodified but ellipsoidally truncated (Zebisch et al., 2012) diffraction data.

Surface Plasmon Resonance

Equilibrium experiments were performed as described before (Zebisch et al., 2013; Kakugawa et al., 2015) with the addition of 2 mM $CaCl_2$ for experiments investigating the direct $LRP6_{PE1PE2}$-$KRM1_{ECD}$ interaction.

ACCESSION NUMBERS

Coordinates and structure factors have been deposited in the PDB with succession numbers PDB: 5FWS, 5FWT, 5FWU, 5FWV, and 5FWW.

SUPPLEMENTAL INFORMATION

Supplemental Information includes one figure and can be found with this article online at http://dx.doi.org/10.1016/j.str.2016.06.020.

AUTHOR CONTRIBUTIONS

M.Z. and V.A.J. performed experiments with support from Y.Z., who generated the stable cell line. M.Z. and E.Y.J. designed the research. M.Z. wrote the paper with input from all other authors.

ACKNOWLEDGMENTS

We thank the staff of Diamond beamlines i03 and i04 for assistance with X-ray diffraction data collection (proposal mx8423), W. Lu for help with tissue culture, and K. Harlos and T. Walter for assistance with crystallization. This work was funded by Cancer Research UK and the UK Medical Research Council (to E.Y.J., A10976 and G9900061) and an IEF Marie Curie fellowship awarded to M.Z. The Wellcome Trust Center for Human Genetics is supported by Wellcome Trust Centre grant 090532/Z/09/Z.

Received: February 23, 2016
Revised: June 12, 2016
Accepted: June 13, 2016
Published: August 11, 2016

REFERENCES

Ahn, V.E., Chu, M.L., Choi, H.J., Tran, D., Abo, A., and Weis, W.I. (2011). Structural basis of Wnt signaling inhibition by Dickkopf binding to LRP5/6. Dev. Cell *21*, 862–873.

Bafico, A., Liu, G., Yaniv, A., Gazit, A., and Aaronson, S.A. (2001). Novel mechanism of Wnt signalling inhibition mediated by Dickkopf-1 interaction with LRP6/Arrow. Nat. Cell Biol. *3*, 683–686.

Bao, J., Zheng, J.J., and Wu, D. (2012). The structural basis of DKK-mediated inhibition of Wnt/LRP signaling. Sci. Signal. *5*, e22.

Bourhis, E., Wang, W., Tam, C., Hwang, J., Zhang, Y., Spittler, D., Huang, O.W., Gong, Y., Estevez, A., Zilberleyb, I., et al. (2011). Wnt antagonists bind through a short peptide to the first beta-propeller domain of LRP5/6. Structure *19*, 1433–1442.

Briggs, D.C., Birchenough, H.L., Ali, T., Rugg, M.S., Waltho, J.P., Ievoli, E., Jowitt, T.A., Enghild, J.J., Richter, R.P., Salustri, A., et al. (2015). Metal ion-dependent heavy chain transfer activity of Tsg-6 mediates assembly of the cumulus-oocyte matrix. J. Biol. Chem. *290*, 28708.

Causeret, F., Sumia, I., and Pierani, A. (2015). Kremen1 and Dickkopf1 control cell survival in a Wnt-independent manner. Cell Death Differ. *23*, 323–332.

Chang, T.H., Hsieh, F.L., Zebisch, M., Harlos, K., Elegheert, J., and Jones, E.Y. (2015). Structure and functional properties of Norrin mimic Wnt for signalling with Frizzled4, Lrp5/6, and proteoglycan. Elife *4*, http://dx.doi.org/10.7554/eLife.06554.

Chen, L., Wang, K., Shao, Y., Huang, J., Li, X., Shan, J., Wu, D., and Zheng, J.J. (2008). Structural insight into the mechanisms of Wnt signaling antagonism by Dkk. J. Biol. Chem. *283*, 23364–23370.

Chen, S., Bubeck, D., Macdonald, B.T., Liang, W.X., Mao, J.H., Malinauskas, T., Llorca, O., Aricescu, A.R., Siebold, C., He, X., et al. (2011). Structural and functional studies of LRP6 ectodomain reveal a platform for Wnt signaling. Dev. Cell *21*, 848–861.

Cheng, Z., Biechele, T., Wei, Z., Morrone, S., Moon, R.T., Wang, L., and Xu, W. (2011). Crystal structures of the extracellular domain of LRP6 and its complex with DKK1. Nat. Struct. Mol. Biol. *18*, 1204–1210.

Clevers, H., and Nusse, R. (2012). Wnt/beta-catenin signaling and disease. Cell *149*, 1192–1205.

Cselenyi, C.S., and Lee, E. (2008). Context-dependent activation or inhibition of Wnt-beta-catenin signaling by Kremen. Sci. Signal. *1*, e10.

Davidson, G., Mao, B., del Barco Barrantes, I., and Niehrs, C. (2002). Kremen proteins interact with Dickkopf1 to regulate anteroposterior CNS patterning. Development *129*, 5587–5596.

Ellwanger, K., Saito, H., Clement-Lacroix, P., Maltry, N., Niedermeyer, J., Lee, W.K., Baron, R., Rawadi, G., Westphal, H., and Niehrs, C. (2008). Targeted disruption of the Wnt regulator Kremen induces limb defects and high bone density. Mol. Cell. Biol. *28*, 4875–4882.

Gaboriaud, C., Gregory-Pauron, L., Teillet, F., Thielens, N.M., Bally, I., and Arlaud, G.J. (2011). Structure and properties of the Ca(2+)-binding CUB domain, a widespread ligand-recognition unit involved in major biological functions. Biochem. J. *439*, 185–193.

Hassler, C., Cruciat, C.M., Huang, Y.L., Kuriyama, S., Mayor, R., and Niehrs, C. (2007). Kremen is required for neural crest induction in *Xenopus* and promotes LRP6-mediated Wnt signaling. Development *134*, 4255–4263.

Holstein, T.W. (2012). The evolution of the Wnt pathway. Cold Spring Harb. Perspect. Biol. *4*, a007922.

Issa, Y.A., Kamal, L., Rayyan, A.A., Dweik, D., Pierce, S., Lee, M.K., King, M.C., Walsh, T., and Kanaan, M. (2016). Mutation of KREMEN1, a modulator of Wnt signaling, is responsible for ectodermal dysplasia including oligodontia in Palestinian families. Eur. J. Hum. Genet. http://dx.doi.org/10.1038/ejhg.2016.29.

Kakugawa, S., Langton, P.F., Zebisch, M., Howell, S.A., Chang, T.H., Liu, Y., Feizi, T., Bineva, G., O'Reilly, N., Snijders, A.P., et al. (2015). Notum deacylates Wnt proteins to suppress signalling activity. Nature *519*, 187–192.

Malinauskas, T., and Jones, E.Y. (2014). Extracellular modulators of Wnt signalling. Curr. Opin. Struct. Biol. *29C*, 77–84.

Mao, B., and Niehrs, C. (2003). Kremen2 modulates Dickkopf2 activity during Wnt/LRP6 signaling. Gene *302*, 179–183.

Mao, B., Wu, W., Li, Y., Hoppe, D., Stannek, P., Glinka, A., and Niehrs, C. (2001). LDL-receptor-related protein 6 is a receptor for Dickkopf proteins. Nature *411*, 321–325.

Mao, B., Wu, W., Davidson, G., Marhold, J., Li, M., Mechler, B.M., Delius, H., Hoppe, D., Stannek, P., Walter, C., et al. (2002). Kremen proteins are Dickkopf receptors that regulate Wnt/beta-catenin signalling. Nature *417*, 664–667.

McCoy, A.J., Grosse-Kunstleve, R.W., Adams, P.D., Winn, M.D., Storoni, L.C., and Read, R.J. (2007). Phaser crystallographic software. J. Appl. Crystallogr. *40*, 658–674.

Nakamura, T., Aoki, S., Kitajima, K., Takahashi, T., Matsumoto, K., and Nakamura, T. (2001). Molecular cloning and characterization of Kremen, a novel kringle-containing transmembrane protein. Biochim. Biophys. Acta *1518*, 63–72.

Nakamura, T., Nakamura, T., and Matsumoto, K. (2008). The functions and possible significance of Kremen as the gatekeeper of Wnt signalling in development and pathology. J. Cell Mol. Med. *12*, 391–408.

Niehrs, C. (2012). The complex world of WNT receptor signalling. Nat. Rev. Mol. Cell Biol. *13*, 767–779.

Osada, M., Ito, E., Fermin, H.A., Vazquez-Cintron, E., Venkatesh, T., Friedel, R.H., and Pezzano, M. (2006). The Wnt signaling antagonist Kremen1 is required for development of thymic architecture. Clin. Dev. Immunol. *13*, 299–319.

Peroutka, R.J., Elshourbagy, N., Piech, T., and Butt, T.R. (2008). Enhanced protein expression in mammalian cells using engineered SUMO fusions: secreted phospholipase A2. Protein Sci. *17*, 1586–1595.

Romero, A., Romao, M.J., Varela, P.F., Kolln, I., Dias, J.M., Carvalho, A.L., Sanz, L., Topfer-Petersen, E., and Calvete, J.J. (1997). The crystal structures of two spermadhesins reveal the CUB domain fold. Nat. Struct. Biol. *4*, 783–788.

Schulze, J., Seitz, S., Saito, H., Schneebauer, M., Marshall, R.P., Baranowsky, A., Busse, B., Schilling, A.F., Friedrich, F.W., Albers, J., et al. (2010). Negative regulation of bone formation by the transmembrane Wnt antagonist Kremen-2. PLoS One *5*, e10309.

Seiradake, E., Zhao, Y., Lu, W., Aricescu, A.R., and Jones, E.Y. (2015). Production of cell surface and secreted glycoproteins in mammalian cells. Methods Mol. Biol. *1261*, 115–127.

Semenov, M.V., Tamai, K., Brott, B.K., Kuhl, M., Sokol, S., and He, X. (2001). Head inducer Dickkopf-1 is a ligand for Wnt coreceptor LRP6. Curr. Biol. *11*, 951–961.

Strong, M., Sawaya, M.R., Wang, S., Phillips, M., Cascio, D., and Eisenberg, D. (2006). Toward the structural genomics of complexes: crystal structure of a PE/PPE protein complex from *Mycobacterium tuberculosis*. Proc. Natl. Acad. Sci. USA *103*, 8060–8065.

Tordai, H., Banyai, L., and Patthy, L. (1999). The PAN module: the N-terminal domains of plasminogen and hepatocyte growth factor are homologous with the apple domains of the prekallikrein family and with a novel domain found in numerous nematode proteins. FEBS Lett. *461*, 63–67.

Verna, J., Lodder, A., Lee, K., Vagts, A., and Ballester, R. (1997). A family of genes required for maintenance of cell wall integrity and for the stress response in *Saccharomyces cerevisiae*. Proc. Natl. Acad. Sci. USA *94*, 13804–13809.

Wang, K., Zhang, Y., Li, X., Chen, L., Wang, H., Wu, J., Zheng, J., and Wu, D. (2008). Characterization of the Kremen-binding site on Dkk1 and elucidation of the role of Kremen in dkk-mediated Wnt antagonism. J. Biol. Chem. *283*, 23371–23375.

Watanabe, K., Chirgadze, D.Y., Lietha, D., de, J.H., Blundell, T.L., and Gherardi, E. (2002). A new crystal form of the NK1 splice variant of HGF/SF demonstrates extensive hinge movement and suggests that the NK1 dimer originates by domain swapping. J. Mol. Biol. *319*, 283–288.

Wu, T.P., Padmanabhan, K.P., and Tulinsky, A. (1994). The structure of recombinant plasminogen kringle 1 and the fibrin binding site. Blood Coagul. Fibrinolysis *5*, 157–166.

Zebisch, M., Krauss, M., Schäfer, P., and Sträter, N. (2012). Crystallographic evidence for a domain motion in rat nucleoside triphosphate diphosphohydrolase (NTPDase) 1. J. Mol. Biol. *415*, 288–306.

Zebisch, M., Xu, Y., Krastev, C., Macdonald, B.T., Chen, M., Gilbert, R.J., He, X., and Jones, E.Y. (2013). Structural and molecular basis of ZNRF3/RNF43 transmembrane ubiquitin ligase inhibition by the Wnt agonist R-spondin. Nat. Commun. *4*, 2787.

Structure, Volume *24*

Supplemental Information

Structure of the Dual-Mode Wnt Regulator Kremen1 and Insight into Ternary Complex Formation with LRP6 and Dickkopf

Matthias Zebisch, Verity A. Jackson, Yuguang Zhao, and E. Yvonne Jones

Structure of the dual mode Wnt regulator Kremen1 and insight into ternary complex formation with LRP6 and Dickkopf

Matthias Zebisch, Verity A. Jackson, Yuguang Zhao & E. Yvonne Jones

Supplementary Information

Inventory of Supplemental Information

Figure S1: Representative electron density for the ternary complex solved at low resolution. Relates to Figure 2.

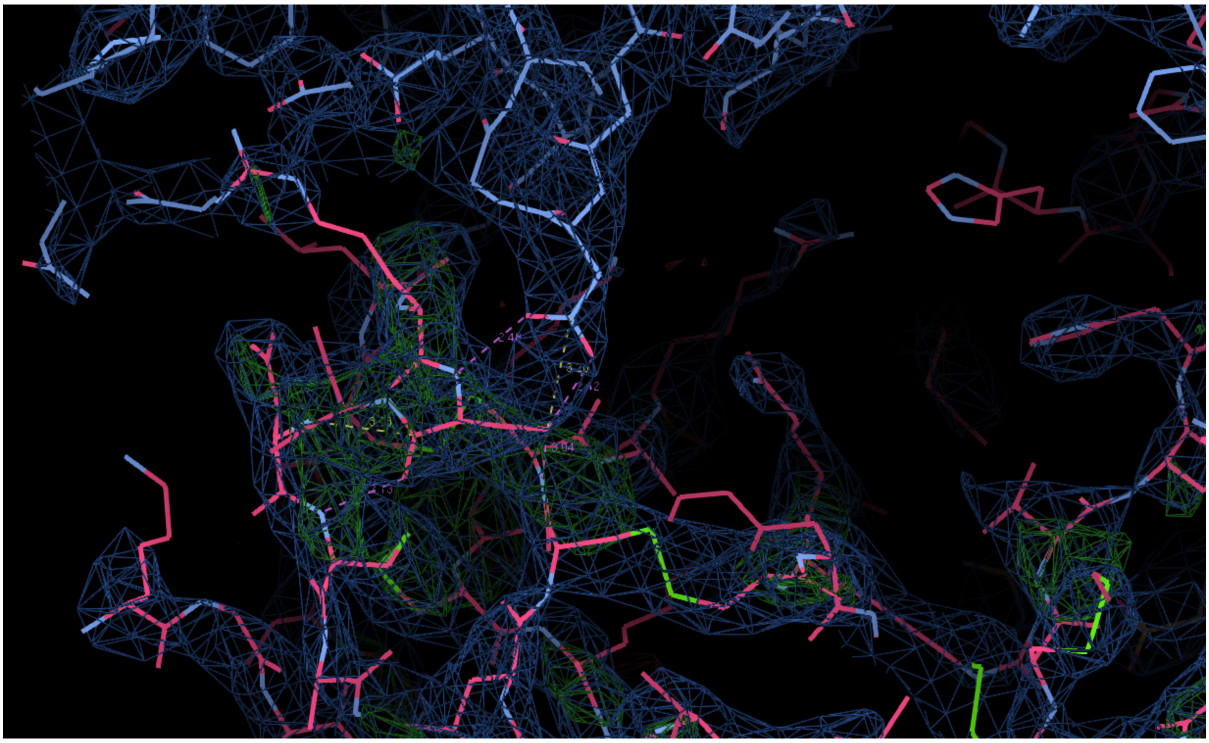

Figure S1: Representative electron density for the ternary complex solved at low resolution. Relates to Figure 2. The KRM1-DKK1 interaction around D201 (KRM1) and S192 (DKK1) is highlighted. Shown is the 2FoFc map calculated for the final model contoured at 1σ in blue and the FoFc DKK1 omit map contoured at 3σ in green.
